# Improving the Robustness of Organic Semiconductors through Hydrogen Bonding

**DOI:** 10.1021/acsami.0c18928

**Published:** 2021-02-12

**Authors:** Paula Gómez, Stamatis Georgakopoulos, Miriam Más-Montoya, Jesús Cerdá, José Pérez, Enrique Ortí, Juan Aragó, David Curiel

**Affiliations:** † Multifunctional Molecular Materials Group, Department of Organic Chemistry, University of Murcia, Campus of Espinardo, 30100 Murcia, Spain; ‡ Institute of Molecular Science, University of Valencia, Catedrático José Beltrán 2, 46980 Paterna, Spain; § Department of Chemical Engineering and Environmental Chemistry, Regional Campus of International Excellence, Technical University of Cartagena, 30203 Cartagena, Spain

**Keywords:** hydrogen-bonded materials, self-assembly, organic semiconductors, hole-transporting materials, organic electronics, charge transport, organic field-effect transistors

## Abstract

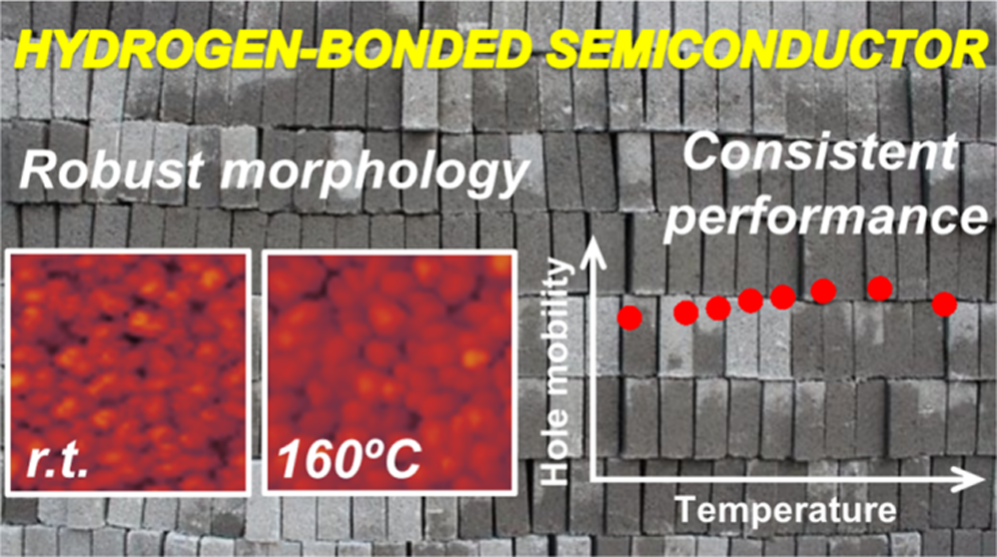

Molecular organization plays an essential role in organic semiconductors since it determines the extent of intermolecular interactions that govern the charge transport present in all electronic applications. The benefits of hydrogen bond-directed self-assembly on charge transport properties are demonstrated by comparing two analogous pyrrole-based, fused heptacyclic molecules. The rationally designed synthesis of these materials allows for inducing or preventing hydrogen bonding. Strategically located hydrogen bond donor and acceptor sites control the solid-state arrangement, favoring the supramolecular expansion of the π-conjugated surface and the subsequent π-stacking as proved by X-ray diffraction and computational calculations. The consistency observed for the performance of organic field-effect transistors and the morphology of the organic thin films corroborate that higher stability and thermal robustness are achieved in the hydrogen-bonded material.

## Introduction

Organic semiconductors have gained much interest due to their application in the areas of electronics and photonics for the fabrication of a new generation of optoelectronic devices such as organic light-emitting diodes (OLEDs), organic field-effect transistors (OFETs), or organic and hybrid solar cells, among other products related to electronic applications.^1−4^ The technical properties of this new generation of devices (flexibility, lightweight, and reduced cost) make them very appealing for the electronics industry. Nevertheless, although devices such as OLEDs have already reached the stage of commercial exploitation,^5^ some issues mainly related to the stability, durability, and efficiency of organic materials, and devices based on these types of materials, still need to be solved so that organic electronics can truly represent a profitable alternative.^6^

An important part of the typical limitations of organic semiconductors comes from their nature as molecular solids. The solid-state arrangement of these materials is mainly governed by weak noncovalent intermolecular interactions, which is the reason why organic semiconductors are commonly considered as disordered solids.^7,8^ As a consequence, wide electronic band structures, comparable to those found for inorganic semiconductors, are not generally achieved, and bandlike transport is rather uncommon in organic semiconductors.^9,10^ This has been observed in highly pure crystals at low temperatures where molecular vibrations are to a large extent quenched.^11−14^ Nevertheless, charge transport in disordered organic thin films at room temperature becomes more difficult^15,16^ because it has to follow a thermally activated hopping mechanism within a manifold of localized states.^17−19^ These localized states are associated with the frontier molecular orbitals of the organic semiconductor, whose topology and overlap between neighboring molecules determine the charge transport.^20^ As such, the molecular organization in the solid state strongly affects charge mobility and thus electrical performance.^21,22^ Given the π-conjugated structure of organic semiconductors, the connection between molecules is mainly due to π–π interactions that, according to the relative orientation of the interacting molecules, could be basically classified as either face-to-face or edge-to-face.^23^ Despite the indispensable contribution of these π–π interactions to charge carrier hopping, especially in the case of face-to-face stacking, it is difficult to adjust their direction with the aim of gaining some control on the molecular organization in the solid state.^24^ Hence, the cooperative effect of stronger noncovalent interactions could enable better control over the disposition of π-conjugated molecules. In this regard, hydrogen bonding is particularly helpful.^25,26^ The large variety of functional groups and building blocks that can work as hydrogen bond donor or hydrogen bond acceptor units becomes a synthetic resource that opens up the possibility of preparing an enormous diversity of molecules. Additionally, the high energy of hydrogen bonds, from the perspective of noncovalent interactions, as well as their directionality, would result in more stable materials where the orientation of the molecules could be better predicted. This strategy based on hydrogen bond-directed self-assembly has been widely used to control the molecular arrangement in the solid state, and also in solution, through the discipline of crystal engineering, representing one of the most important tools for the development of supramolecular chemistry.^27−30^ Moreover, the macromolecular organization through hydrogen bond interactions is ubiquitous in biological systems.^31^

In agreement with these premises, the approach based on molecular self-assembly could also contribute to the development of novel semiconducting materials and the progress of organic electronics. Although this methodology has been explored for some conjugated materials, the synthetic strategy often turns to the peripheral functionalization of the conjugated system with hydrogen bonding functions and solubilizing groups.^31−33^ However, the bulkiness and insulating character of the attached substituents could interfere with the charge transport process. Only recently, conjugated systems integrating hydrogen bond donor and acceptor sites within the conjugated structure have been reported. In this context, it is worth highlighting the excellent results obtained for different studies of quinacridone and epindolidione leading to high hole mobilities in thin-film OFETs, even showing ambipolar charge transport by means of interfacial engineering.^34^ Analogously, the building block of indolinone admits different isomer structures whose hydrogen bonding properties can be exploited in the field of organic electronics. This is the case of the studies reported for indigo and diketopyrrolopyrroles.^35−37^ Additionally, an elegant tuning of the molecular arrangement *via* hydrogen bonding was achieved through the perchlorination of perylenediimide, which reached quite remarkable electron mobility upon controlling the substrate temperature during the OFET fabrication.^38^ All of these examples have in common the use of N–H groups as hydrogen bond donors and carbonyl groups as hydrogen bond acceptors. Some other structural motifs that have been reported to form hydrogen-bonded frameworks in organic semiconductors include pentacene and hexacene quinones (C–H···O=C),^39^ dichloropentacene (C–H···Cl),^40^ dihydrotetraazapentacenes (N–H···N),^41^ and pyrrolobenzothiazines (N–H···N, C–H···N).^42^

**Scheme 1:**
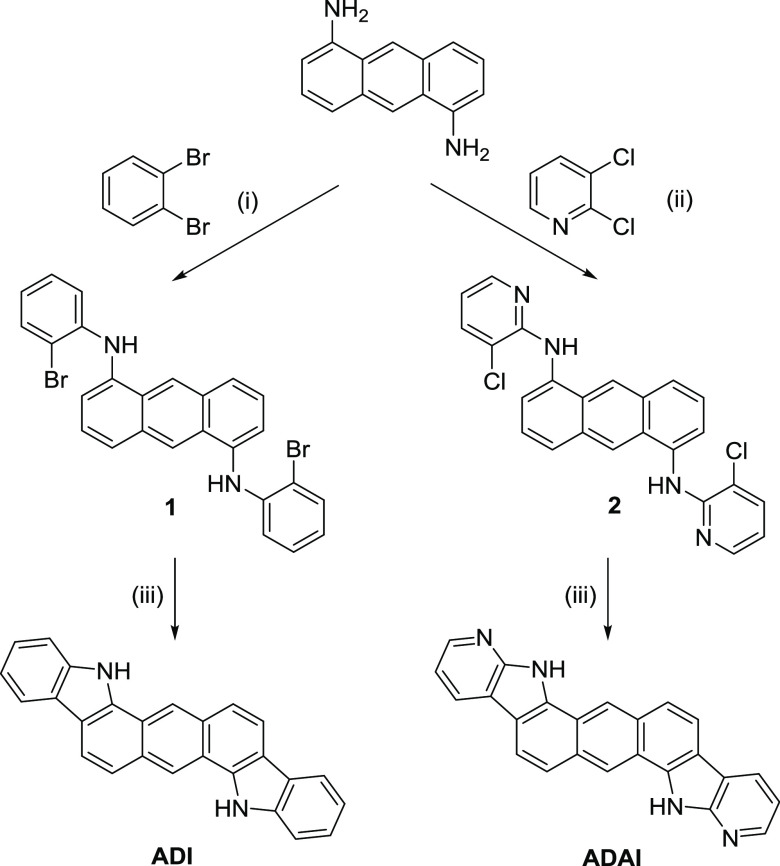
Synthetic Routes for **ADI** and **ADAI** (i) (±)-2,2′-Bis(diphenylphosphino)-1,1′-binaphthalene (BINAP), Pd(dba)_2_, K_2_CO_3_, toluene, reflux (37%); (ii) (±)-BINAP, Pd(OAc)2, *t*BuOK, 1,4-dioxane, reflux (68%); and (iii) dimethyl sulfoxide (DMSO), *t*BuOK, *h*ν (**ADI**: 67%; **ADAI**: 96%).

Recently, we have reported the synthesis of a series of molecules where the integration of the 7-azaindole substructure into a fused polyheteroaromatic system results in a hydrogen bond-directed self-assembly (N–H_pyrrole_···N_pyridine_).^43,44^ Interestingly, this type of structure does not compromise the conjugation throughout the whole fused polycyclic molecule, allowing for better charge delocalization. We hypothesized with the possibility that, upon self-assembly, the supramolecular adduct would present an extended π-surface that subsequently would improve the intermolecular π–π interactions. Based on this hypothesis, the increase of the conjugation length in the molecule to self-assemble should further benefit the intermolecular interactions. Thus, we present a comparative study between two heptacyclic compounds, namely, 7,15-dihydroanthra[1,2-*b*:5,6-*b*′]diindole, **ADI**, and 7,15-dihydroanthra[1,2-*b*:5,6-*b*′]di(7-azaindole), **ADAI** (Scheme 1). The benzene or pyridine rings located at the extremes of each molecule (**ADI** or **ADAI**) make a critical difference that, respectively, prevents or enables the intermolecular interactions by hydrogen bonding (N–H_pyrrole_···N_pyridine_). As a consequence, noticeable differences are observed in the solid-state packing. This affects the thin-film morphology and hole mobility, as further evidenced by observing the evolution upon thermal annealing of the thin-film transistors fabricated with the reported materials. The significant robustness observed for the self-assembled material based on **ADAI** demonstrates the suitability of our self-assembly strategy for the design of π-conjugated systems, conferring high stability to the organic thin films and the corresponding electronic devices.

## Experimental Section

### General

Commercially available reagents and solvents were used for the synthesis, purification, and characterization of the reported compounds without any additional purification unless stated otherwise. **ADI** and **ADAI** were obtained with high purity by purification *via* gradient sublimation (pressure < 10^–6^ mbar). ^1^H NMR and ^13^C NMR spectra were acquired on a Bruker AV200, Bruker AV300, or Bruker AV400 spectrometer. The residual solvent peaks were used as a reference for the chemical shifts, which are given in ppm. Mass spectra were measured on a high-performance liquid chromatography-mass spectrometry (HPLC-MS) time-of-flight (TOF) 6220 instrument. Noncorrected melting points were measured using a Reichert instrument. An SDT 2960 analyzer from TA Instruments was employed for thermogravimetric analysis (TGA) under an inert atmosphere (heating rate: 10 °C min^–1^). Ultraviolet–visible (UV–vis) spectra were acquired on a Cary 5000 spectrophotometer using dimethylformamide (DMF) solutions. Cyclic voltammetry experiments were performed in DMF using a BAS potentiostat. A boron-doped diamond was used as the working electrode, Pt wire was used as the counter electrode, Ag/AgCl as the reference electrode, and the ferrocene/ferrocenium (Fc/Fc^+^) couple as the internal reference. The scan rate was 100 mV s^–1^. Tetrabutylammonium hexafluorophosphate (0.1 M) was the supporting electrolyte.

### Synthetic Methods

Compound **2** and **ADAI** were synthesized following previously reported synthetic procedures.^44^

#### *N*,*N*′-Bis(2-bromophenyl)anthracene-1,5-diamine, **1**

(±)-2,2′-Bis(diphenylphosphino)-1,1′-binaphthalene (BINAP) (0.22 g, 7.5 mol%) was suspended in distilled toluene (30 mL) in a two-neck round-bottom flask under a nitrogen atmosphere. The mixture was heated in an oil bath until it was completely dissolved. Then, bis(dibenzylideneacetone)palladium(0) (Pd(dba)_2_) (0.14 g, 5.0 mol%) was added, and the resulting solution was stirred for 15 min. In another two-neck round-bottom flask under an inert atmosphere, 1,5-diaminoanthracene (1.00 g, 4.80 mmol), 1,2-dibromobenzene (1.74 mL, 14.4 mmol), and potassium carbonate (3.98 g, 28.8 mmol) were placed with 30 mL of distilled toluene. The catalyst was transferred *via* a syringe to the reaction flask and it was heated at the reflux temperature. The reaction evolution was followed using thin layer chromatography. Finally, the crude was cooled to room temperature, and the solvent was removed under reduced pressure. The resulting crude was washed with water (3 × 30 mL) and triturated with diethyl ether (3 × 30 mL) to obtain the *N*-arylated compound (1.86 g, 37%). Mp.: 233–234 °C. ^1^H NMR (400 MHz, CDCl_3_), δ: 8.64 (s, 2H), 7.84 (dd, *J* = 6.4, 3.2 Hz, 2H), 7.60 (dd, *J* = 8.4, 1.6 Hz, 2H), 7.43 (t, *J* = 6.4 Hz, 2H), 7.43 (d, *J* = 3.2 Hz, 2H), 7.10 (td, *J* = 8.4, 1.2 Hz, 2H), 6.96 (dd, *J* = 8.4, 1.2 Hz, 2H), 6.75 (td, *J* = 8.4, 1.6 Hz, 2H), 6.52 (s, 2H, NH) ppm. ^13^C NMR (50.3 MHz, CDCl_3_), δ: 142.9, 137.3, 132.8, 132.7, 128.2, 128.0, 125.5, 125.4, 121.7, 120.5, 118.1, 116.1, 111.6 ppm. HRMS (ESI) *m*/*z*: [M + H]^+^ Calcd. for C_26_H_19_Br_2_N_2_: 516.991; Found: 516.9901.

#### 7,15-Dihydroanthra[1,2-*b*:5,6-*b*′]diindole, **ADI**

To a photochemical reactor containing anhydrous dimethyl sulfoxide (160 mL) under a continuous nitrogen flow, potassium *tert*-butoxide (1.34 g, 11.9 mmol) and *N*,*N*′-bis(2-bromophenyl)anthracene-1,5-diamine (1.41 g, 2.72 mmol) were sequentially added. The resulting mixture was degassed for 15 min before being irradiated with a medium-pressure mercury lamp for 2.0 h. Afterward, the crude was allowed to reach room temperature and poured into a saturated solution of ammonium chloride (50 mL). The resulting precipitate was filtered under vacuum, washed with water (3 × 30 mL) and methanol (3 × 30 mL) to isolate the desired product (0.65 g, 67%). Mp: > 300 °C. ^1^H NMR (300 MHz, DMSO-*d*_6_), δ: 12.49 (s, 2H, NH), 9.21 (s, 2H), 8.26 (d, *J* = 8.7 Hz, 2H), 8.19 (d, *J* = 7.7 Hz, 2H), 7.82 (d, *J* = 8.7 Hz, 2H), 7.69 (d, *J* = 7.7 Hz, 2H), 7.41 (t, *J* = 7.7 Hz, 2H), 7.41 (t, *J* = 7.7 Hz, 2H) ppm. ^13^C NMR (101 MHz, DMSO-*d*_6_), δ: 138.9, 135.4, 130.5, 124.6, 124.0, 121.2, 120.7, 120.4, 119.9, 119.9, 119.8, 116.3, 112.0 ppm. HRMS (ESI) *m*/*z*: [M + H]^+^ Calcd. for C_26_H_17_N_2_: 355.1241; Found: 355.1251.

### OFET Fabrication and Characterization

Thin-film OFETs were fabricated with a bottom-gate top-contact configuration. P-doped n^++^ silicon wafers coated with a 300 nm thick layer of thermally grown SiO_2_ were used. The cleaning protocol of the substrates consisted of sequential sonication with water, acetone, and isopropanol for 20 min. Next, a self-assembled monolayer of octadecyltrimethoxysilane (OTS) was grown on the surface of SiO_2_ following a reported protocol.^45^ Then, 50 nm thick films of **ADI** and **ADAI** were thermally evaporated under high vacuum conditions (1 × 10^–7^ mbar) onto the OTS-coated substrates at a rate of 0.2 Å s^–1^. The device structures were completed by the sequential evaporation of 8 nm of MoO_3_ (rate: 0.1 Å s^–1^) and 25 nm of Au (rate: 0.2 Å s^–1^) through a shadow mask. The OFET channel dimensions were 2 mm width and 40–140 μm length confirmed by profilometry. A Keithley 2636A semiconductor parameter analyzer was used to measure the current–voltage (*I*–*V*) curves at room temperature under an ambient atmosphere. The field-effect mobility was extracted in the saturation regime. Before each measurement, the device was annealed in a hot plate at the indicated temperature for 15 min and cooled to room temperature.

### Morphology

Tapping mode atomic force microscopy (AFM) images were recorded using an NT-MDT microscope (NTEGRA PRIMA) and analyzed with Gwyddion V2.47.

### X-ray Diffraction (XRD)

The single-crystal X-ray data were collected at 100 K with a Bruker D8Quest Kappa diffractometer using Cu Kα radiation. The structure was solved using direct methods and refined on F2 by the full-matrix least-squares method, using SHELX-2018 software package and expanded using Fourier techniques. All nonhydrogen atoms were refined anisotropically. The H atom bonded to the nitrogen atom was located in a difference Fourier map and refined freely. Other hydrogen atoms were included in geometrically calculated positions and were refined according to the riding model. CCDC deposition numbers: **ADI** (2036011) and **ADAI** (1957368).

X-ray diffraction diffractograms of the material deposited as a thin film were collected on a Bruker D8 Advance instrument using θ–θ mode with Cu Kα radiation (wavelength 1.54060 Å), 40 kV, 30 mA, and a one-dimensional detector with a window of 1°. Primary optics consisted of a 2° Soller slit, a 1 mm incidence slit, and an air scatter screen. Secondary optics included a 3 mm antiscatter slit, a Ni filter, and a 2.5° Soller slit. The sample was step-scanned from 3 to 65° in 2θ, with 0.05° stepping intervals, 1 s per step, and a rotation speed of 30 rpm.

### Computational Methods

All DFT calculations were carried out within the Gaussian 16 (revision A.03) software package^46^ using the B3LYP-D3BJ^47−49^ functional together with the 6-31G** basis set.^50^*C*_*i*_-symmetry constraints were applied during the geometry optimizations of the neutral and radical cation species to evaluate the internal reorganization energies. Solvent effects were described using the polarized continuum model (PCM) approach.^51^ Transfer integrals *t*_*ij*_ were calculated within the DIPRO approximation^52^ implemented in a homemade program. See the Supporting Information for a full description of the calculation of the charge transport properties.

## Results and Discussion

### Synthesis and Characterization

The synthesis of **ADI** and **ADAI** was accomplished by a straightforward methodology of two steps only (Scheme 1), which represents an important advantage for the simplification of material production. Initially, 1,5-diaminoanthracene^53^ is reacted with the corresponding dihaloarene to obtain the palladium-catalyzed C–N coupling *via* a Buchwald–Hartwig reaction.^54^ In the case of **ADI**, *o*-dibromobenzene was employed. Analogously, 2,3-dichloropyridine was used for the synthesis of **ADAI**. As expected, the latter reacted with high selectivity through position 2 of the pyridine ring to obtain the desired product. The second step consisted of double intramolecular C–C coupling photochemically achieved through a radical aromatic substitution. This synthetic route led to higher yields in the case of the reactions carried out with pyridine than those corresponding to the benzene-ended products.

**Figure 1:**
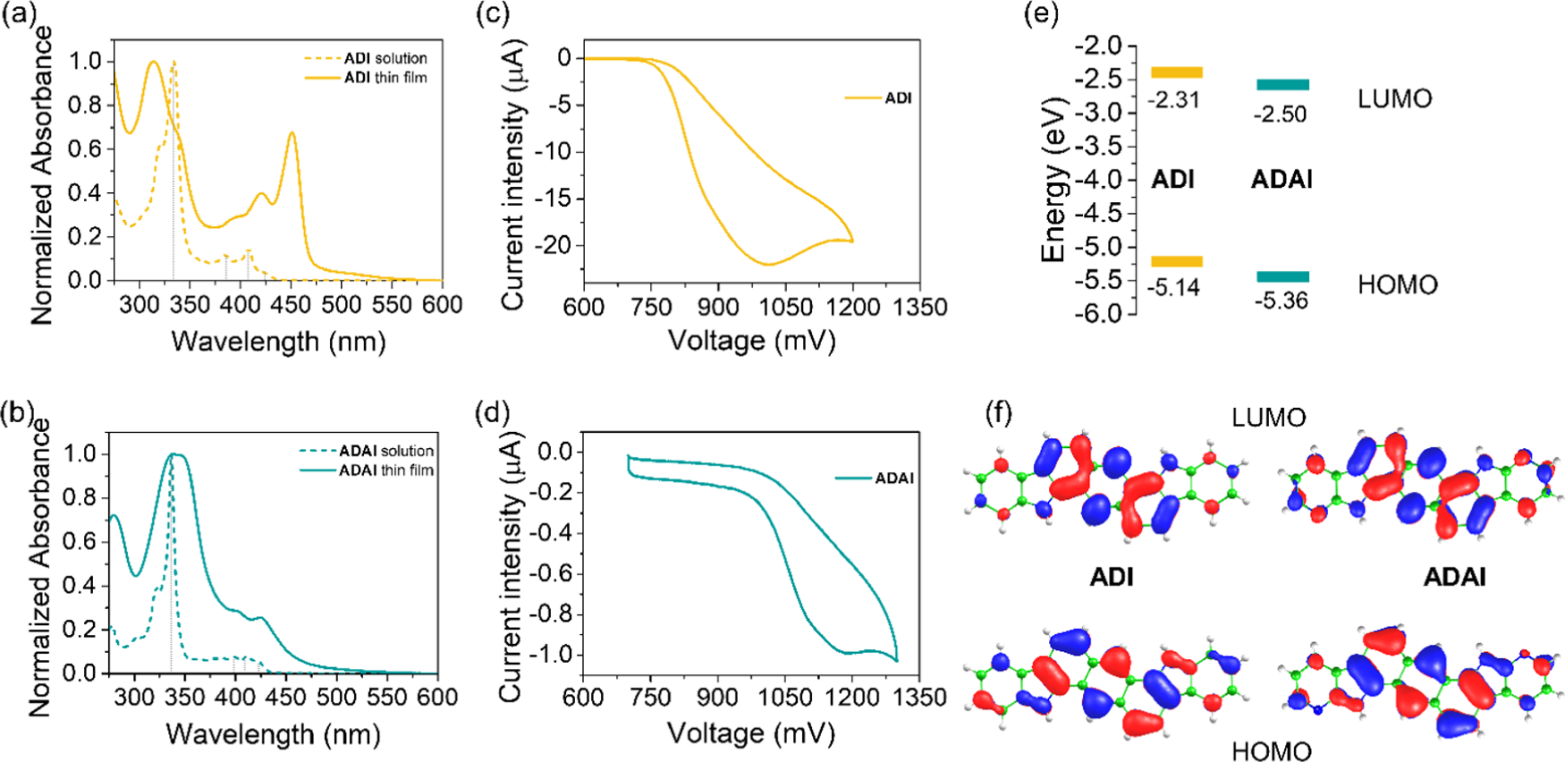
Normalized absorption spectra of (a) **ADI** and (b) **ADAI** in DMF solution and as thin films deposited on quartz substrates. Cyclic voltammograms of (c) **ADI** and (d) **ADAI**. (e) Highest occupied molecular orbital (HOMO) and lowest unoccupied molecular orbital (LUMO) energy levels. (f) Topology of the calculated HOMO and LUMO frontier molecular orbitals.

The electronic properties of the molecular systems were investigated using absorption spectroscopy and cyclic voltammetry. The similarities between both conjugated compounds, **ADI** and **ADAI**, are displayed in the UV–vis spectra (Figure 1a,b) showing the more intense band at 334 and 337 nm, respectively, in dimethylformamide (DMF) solution. Moreover, less intense absorption, ascribed to the α and p bands of the polyheteroaromatic skeleton,^55^ is observed at lower energies, with the spectra onset located at 437 nm (**ADI**) and 433 nm (**ADAI**). Regarding the characterization in the solid state, thin films were evaporated on quartz substrates. The absorption spectra experienced the expected red shift resulting from the intermolecular interactions that narrow the optical gap.

Cyclic voltammograms displayed irreversible electrochemical processes for both **ADI** and **ADAI** compounds (Figure 1c,d), presumably due to poor stability of the radical cation formed after the oxidative scan. The anodic peak potentials (*E*_p-ADI_ = 973 mV; *E*_p-ADAI_ = 1176 mV) evidenced the effect of the more electron-deficient pyridine rings in **ADAI** when compared to those of **ADI** with benzene rings at both extremes of the molecule. Therefore, since the presence of pyridine makes the compound more difficult to be oxidized, a higher potential is measured for **ADAI** (Table 1). In agreement with this, its highest occupied molecular orbital (HOMO) becomes more stable than that determined for **ADI** (Figure 1e). Due to the impossibility of detecting the reduction potential of the studied molecules within the electrochemical window accessible under the employed experimental conditions, the energies of the lowest unoccupied molecular orbital (LUMO) were indirectly estimated by adding the previously measured optical gap to the HOMO energy. It is worth noticing that the low HOMO energies assessed for these compounds could improve their stability toward ambient conditions (atmospheric oxygen and moisture) that commonly cause degradation of electronic devices fabricated with organic materials.

**Table 1:** Optical and Electrochemical Characterization

	λ_onset_^a^ (nm)	*E*_opt._ (eV)	λ_onset_^b^ (nm)	*E*_opt._ (eV)	*E*_onset ox._ (mV)	HOMO^c^ (eV)	LUMO^d^ (eV)
**ADI**	437	2.83	471	2.63	776	–5.14	–2.31
**ADAI**	433	2.86	463	2.68	1000	–5.36	–2.50

a Spectra acquired in DMF solution.

b Spectra for thin films.

c *E*_HOMO_ = −*e*(*E*_onset ox._ – *E*_onset ox. Fc/Fc^+^_) + *E*_HOMO Fc_ (Fc stands for ferrocene; *E*_onset ox. Fc/Fc+_ = 0.44 V; *E*_HOMO Fc_ = −4.8 eV).

d *E*_LUMO_ = *E*_HOMO_ + *E*_opt_.

To obtain further insight into the electronic structures of the **ADI** and **ADAI** systems, density functional theory (DFT) calculations were performed at the B3LYP/6-31G** level in the presence of DMF. Figure 1f displays the topology of the calculated HOMO and LUMO levels. In both cases, the molecular orbitals, although showing a larger contribution from the central anthracene unit, are spread all over the conjugated backbone, which should favor the intramolecular charge delocalization and the intermolecular electronic communication. In terms of energetics, DFT calculations predict an energy stabilization of both HOMO and LUMO levels when passing from **ADI** (−4.95 and −1.60 eV, respectively) to **ADAI** (−5.12 and −1.79 eV). The relative energy shifts of the HOMO and LUMO levels between both systems are in line with the experimental estimates, highlighting that **ADI** exhibits a slightly larger electron-donor character compared to **ADAI**.

The thermal stabilities of **ADI** and **ADAI** were determined by thermogravimetric analysis (Figure S3) that revealed a degradation temperature (*T*_deg._ measured for a 5% mass loss) of 409 °C (**ADI**) and 496 °C (**ADAI**). Although both molecules are considered highly stable, the significant 87 °C difference observed between the *T*_deg._ values can be interpreted as evidence of the higher thermal robustness of **ADAI**. This is very likely due to the solid-state arrangement, stabilized by hydrogen bond interactions, as discussed below.

### Solid-State Structure

**Figure 2:**
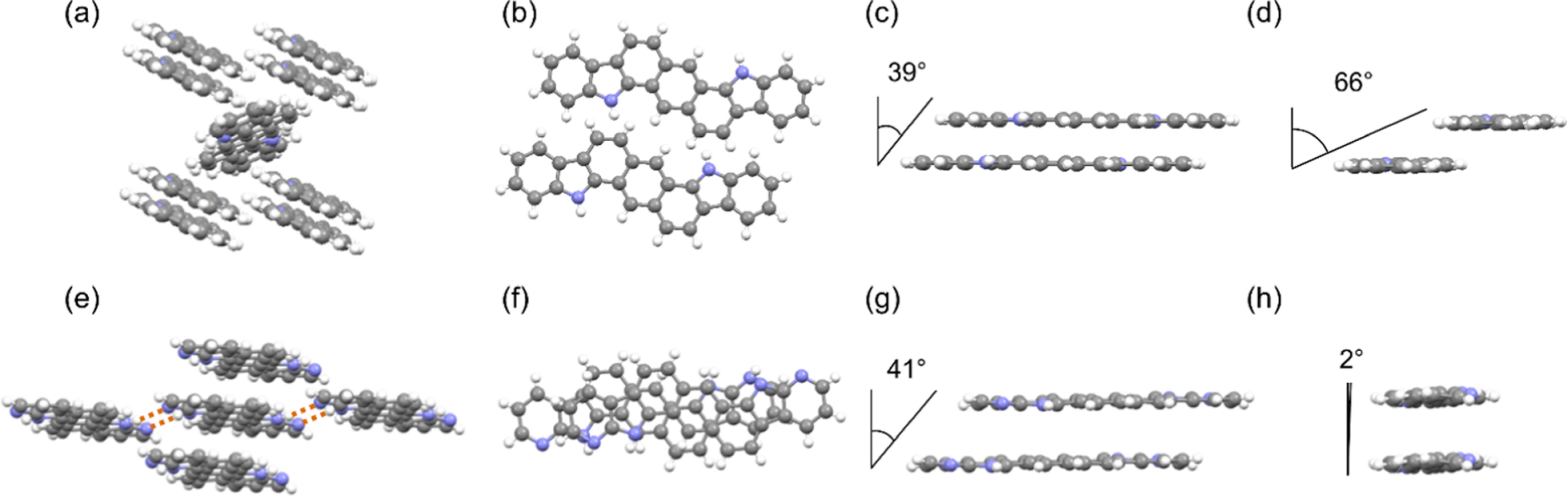
Molecules showing van der Waals contacts in the crystal packing of (a) **ADI** and (e) **ADAI** (color code: gray = carbon, purple = nitrogen, and white = hydrogen), (b) and (f) top view, (c) and (g) view across the short edge, and (d) and (h) view across the long edge of the molecules closely packed in parallel planes for **ADI** and **ADAI**, respectively.

As previously stated, the molecular organization of π-conjugated molecules plays a crucial role in the charge transport ability of organic semiconductors since it conditions the intermolecular orbital overlap that, in the end, directs the charge carrier hopping. X-ray diffraction (XRD) experiments corroborated the expected differences in the crystal packing of **ADI** and **ADAI**. The molecule that cannot self-assemble by hydrogen bonds, *i.e*., **ADI**, crystalizes in a monoclinic cell (space group *P*2_1_/*m*). It shows a herringbone packing, very common in fused polyheteroaromatic systems with a similar structure (Figure 2a).^56,57^

Each molecule of **ADI** is in van der Waals contact with eight neighbor molecules establishing edge-to-face (C–H···π and N–H···π) and face-to-face interactions (π–π). Molecules packed in parallel planes commonly define the orientation where the better overlap is achieved. Nevertheless, a perfect overlap of π-conjugated systems is energetically disfavored due to the electrostatic repulsion between two confronted surfaces with identical electronic densities. Accordingly, fused polyaromatic molecules packed in parallel planes generally adopt a shifted disposition that is characterized by the pitch and roll angles, related to the intermolecular sliding along the long and short molecular edges, respectively.^58^ Taking the longer molecular axis as a reference, **ADI** (Figure 2c,d) shows a pitch angle of 39° and a roll angle of 66°, which, respectively, correspond to a short shift (1.9 Å) along the long edge and a rather long shift (5.4 Å) along the short edge. These shifts determine the intermolecular overlap (Figure 2b), which, in turn, might affect the charge transport.

Regarding the crystal structure of **ADAI**, it crystallizes in a triclinic cell (space group *P* 1̅). The crystal packing is characterized by a slipped-stacking where each molecule is in van der Waals contact with four neighboring molecules (Figure 2e), showing a higher density than that of the crystals of **ADI**. In this case, the centrosymmetric geometry of the molecules leads to a hydrogen bond-directed self-assembly (N–H_pyrrole_···N_pyridine_) that results in a ribbonlike structure. This supramolecular organization contributes to the stabilization of a π-expanded surface. Consequently, these ribbons pile up forming a columnar arrangement or, in other words, the columnar arrangement is stabilized by the hydrogen bonds between adjacent columns (Figure 2e). The observed crystal structure proves our initial hypothesis and reinforces the relevance of hydrogen bonding as a useful tool for the molecular design and the subsequent control of the solid-state structure. **ADAI** molecules packed through π–π stacking display pitch and roll angles of 41° and 2°, respectively (Figure 2g,h). Accordingly, the **ADAI** molecules show short slippage along the long edge (2.9 Å) and short edge (0.1 Å) that is shown in the abovementioned columnar disposition.

### Charge Transport in OFETs and Thin-Film Characterization

**Figure 3:**
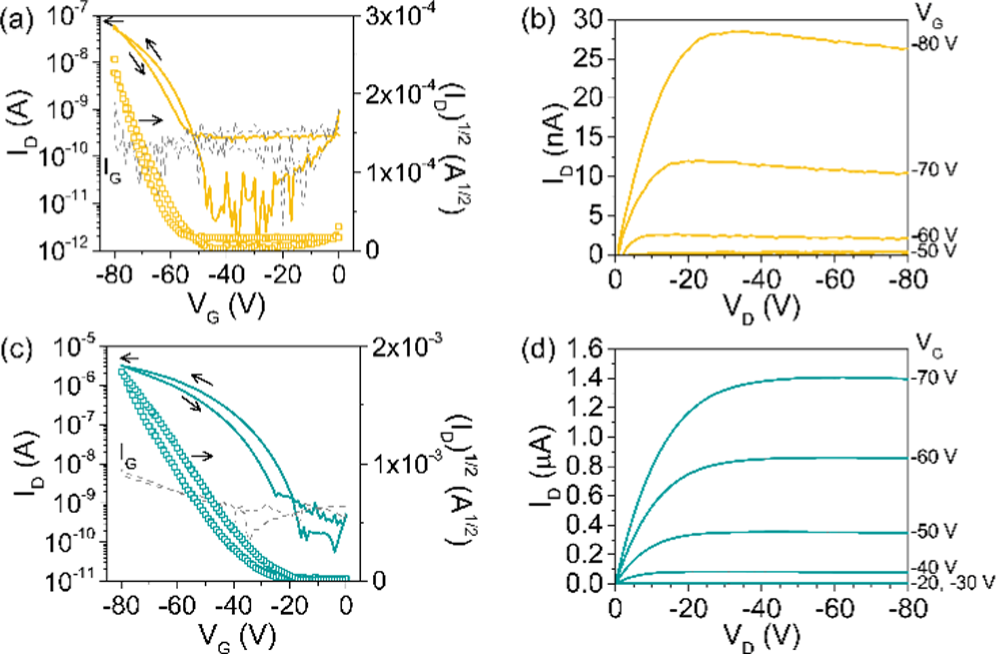
Transfer characteristics of (a) **ADI** and (c) **ADAI** for *V*_D_ = −60 V; dashed lines represent the leakage current. Output characteristics of (b) **ADI** and (d) **ADAI**.

Charge transport properties of **ADI** and **ADAI** were characterized in OFETs with a bottom-gate top-contact architecture, fabricated, without substrate heating, on silicon substrates with a thermally grown silicon oxide layer that was covered with a self-assembled monolayer of OTS. The organic semiconductors were evaporated through a shadow mask, followed by the evaporation of a thin layer of molybdenum oxide and finally the gold contacts. The transfer and output characteristics are depicted in Figure 3. A perfectly linear current intensity–voltage (*I*–*V*) plot is observed at low source–drain voltages (*V*_D_) in the output characteristics, demonstrating the good ohmic contact between the semiconductor and the source–drain electrodes, as it was intended using the MoO_3_ interfacial layer. The high work function of MoO_3_ improves the charge injection on semiconductors with low-lying HOMOs such as **ADI** and **ADAI**.^59^ Mobilities were calculated in the saturated regime from the transfer characteristics measured in air. The **ADI** mobility (4 × 10^–4^ cm^2^ V^–1^ s^–1^) was more than 1 order of magnitude lower than the mobility obtained for the self-assembled **ADAI** (8 × 10^–3^ cm^2^ V^–1^ s^–1^). Moreover, the switch-on voltage (*V*_ON_) in the transistors fabricated with **ADI** (−48 V) was significantly higher than that measured for **ADAI** (−16 V). This can be ascribed to the presence of a lower density of charge traps in the case of the hydrogen-bonded semiconductor, whose transistors showed much better performance.^60^

**Figure 4:**
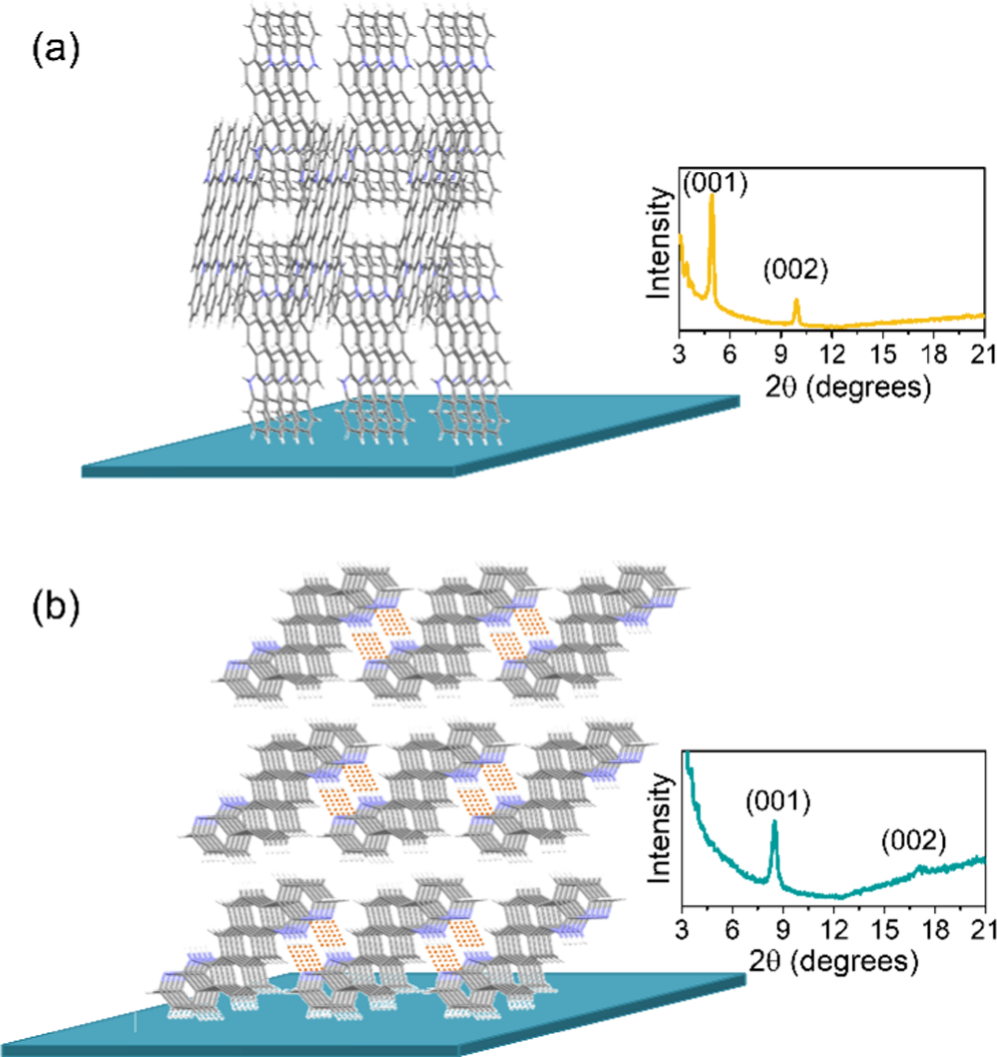
Idealized representation of the molecular arrangement on the OFET substrate surface for (a) **ADI** and (b) **ADAI**. The inset shows the XRD diffractograms.

The organic thin films deposited on the OFET substrates were also characterized using out-of-plane X-ray diffraction (Figure 4). The powder diffraction pattern showed a good correlation with that obtained from the XRD data of the above-discussed solids. Therefore, the molecular packing in the films is consistent with that previously depicted in Figure 2. Besides, the diffractograms showed only a set of periodic peaks, indicating the existence of a prevalent crystalline phase in the thin films. Accordingly, taking the indexation of the crystalline planes and assuming that the out-of-plane reflections correspond to planes parallel to the substrate, we can get an approximation of how the molecules are oriented on the substrate surface (Figure 4).^61^ As far as **ADI** molecules are concerned, they adopt an edge-on orientation, standing on their short edge, with their long edge almost orthogonal to the substrate surface. The calculated *d*-spacing (17.85 Å; 2θ *=* 4.95°) fits well with this vertical alignment since it is very close to the length of the molecule (16.56 Å). Differently, although **ADAI** molecules also arrange in an edge-on disposition, the driving force of the hydrogen-bonded self-assembly affects the orientation of the ribbonlike supramolecular ensemble with respect to the substrate. In agreement with this, the long edge of the molecule is more tilted. This leaned positioning is supported by the calculated *d*-spacing (5.23 Å; 2θ = 8.49°), which is much shorter than the length of the molecule (16.39 Å).

### Theoretical Charge Transport Properties of the Crystals

To get a more complete understanding of the charge transport properties of the **ADI** and **ADAI** semiconductors, a computational protocol, which combines quantum-chemical calculations (DFT), molecular dynamics (MD) simulations, an analytical Marcus–Levich–Jortner (MLJ) electron-transfer rate expression,^62−64^ and a master equation approach, was employed to predict hole mobilities assuming an incoherent regime (see the Supporting Information for full details).

Prior to assessing the MLJ electron-transfer rates (*k*_*ij*_) and mobilities (μ), it is interesting to discuss the average transfer integrals (⟨*t*_*ij*_⟩) and their fluctuations (σ_*t*_*ij*__) due to thermal motions for the different molecular crystal pairs. Note that electronic coupling fluctuations have turned out to be crucial to accurately describe charge and energy transport phenomena in molecular aggregates or crystals.^65−67^ For the **ADI** crystal, only four molecular pairs with short intermolecular contacts hold different ⟨*t*_*ij*_⟩ values (Figure S4 and Table S1). As expected, ⟨*t*_*ij*_⟩ calculated for the parallelly displaced π-stacking dimers (⟨*t*_1,10_⟩) with face-to-face π–π interactions is found to present the highest average value (37.7 meV) with a standard deviation (σ_*t*_1,10__) of 14.5 meV. The edge-to-face molecular dimers display ⟨*t*_1,2_⟩ ± σ_*t*_1,2__ and ⟨*t*_1,6_⟩ ± σ_*t*_1,6__ values of −6.8 ± 16.9 meV and −8.1 ± 10.6 meV, respectively. The relatively large transfer integral fluctuations with respect to the average values are in line with those of other organic semiconducting compounds.^68^

Compared to **ADI**, **ADAI** exhibits more molecular crystal pairs with different and non-negligible ⟨*t*_*ij*_⟩ and σ_*t*_*ij*__ values (Figure S5 and Table S1). These molecular pairs are mainly governed by edge-to-edge (hydrogen bonding) and face-to-face (π–π) interactions without edge-to-face contacts. It should be stressed that the edge-to-edge interactions commonly result in nonexistent overlap, but in this case, the hydrogen bond-directed self-assembly strengthens these interactions and can open up alternative charge transport pathways. For instance, a relatively important average transfer integral and standard deviation of 3.0 ± 2.6 meV (*t*_1,11_ = *t*_1,20_) are found for the hydrogen-bonded molecular dimers. Slipped face-to-face dimers that connect parallel columns provide the highest ⟨*t*_*ij*_⟩ values (⟨*t*_1,8_⟩ = ⟨*t*_1,17_⟩ = −12.3 meV) and (⟨*t*_1,12_⟩ = ⟨*t*_1,19_⟩ = −13.3 meV) with significant fluctuation σ_*t*_*ij*__ values (σ_*t*_1,8__ = σ_*t*_1,17__ = 6.5 meV and σ_*t*_1,12__ = σ_*t*_1,19__ = 10.0 meV) close to the average values. For the eclipsed face-to-face π-stacking dimer, a ⟨*t*_1,2_⟩ value of 0.0 meV is calculated, whereas an important fluctuation of the transfer integral is obtained (σ_*t*_1,2__ = 57.8 meV). This finding can be easily justified by the periodic oscillation and sensitivity of the transfer integral values with the longitudinal and transversal slippages between molecules (Figure S6).^69^ Despite the small average transfer integral, a significant charge-transfer rate for this eclipsed face-to-face π-stacking dimer is expected owing to the significant transfer integral fluctuation.

Besides transfer integrals and fluctuations between the different molecular crystal pairs, the calculation of the reorganization energies (internal and external) and the site energy difference (Δ*E*_*ij*_) is needed to evaluate the MLJ electron-transfer rate constant and, consequently, the hole mobilities within the crystal structure for **ADI** and **ADAI**. Regarding the internal reorganization energy (λ_int_), DFT calculations predict λ_int_ values in the same range for both systems, being smaller for **ADI** (159 meV) than for **ADAI** (183 meV). External reorganization energies (λ_ext_) in organic semiconducting crystals are expected to be lower than λ_int_.^70^ For both **ADI** and **ADAI**, λ_ext_ is set to be 50 meV according to values reported for other similar systems in the literature.^71^ Regarding the site energy difference in the crystals, Δ*E*_*ij*_ deviates from zero due to its dependence on an external electric field (see the Supporting Information). The introduction of an external electric field (*F*) is necessary to evaluate the mobilities by means of the master equation from the charge drift velocity. The selected *F* (1 × 10^5^ V cm^–1^) is set according to values reported in the literature.^72^

**Figure 5:**
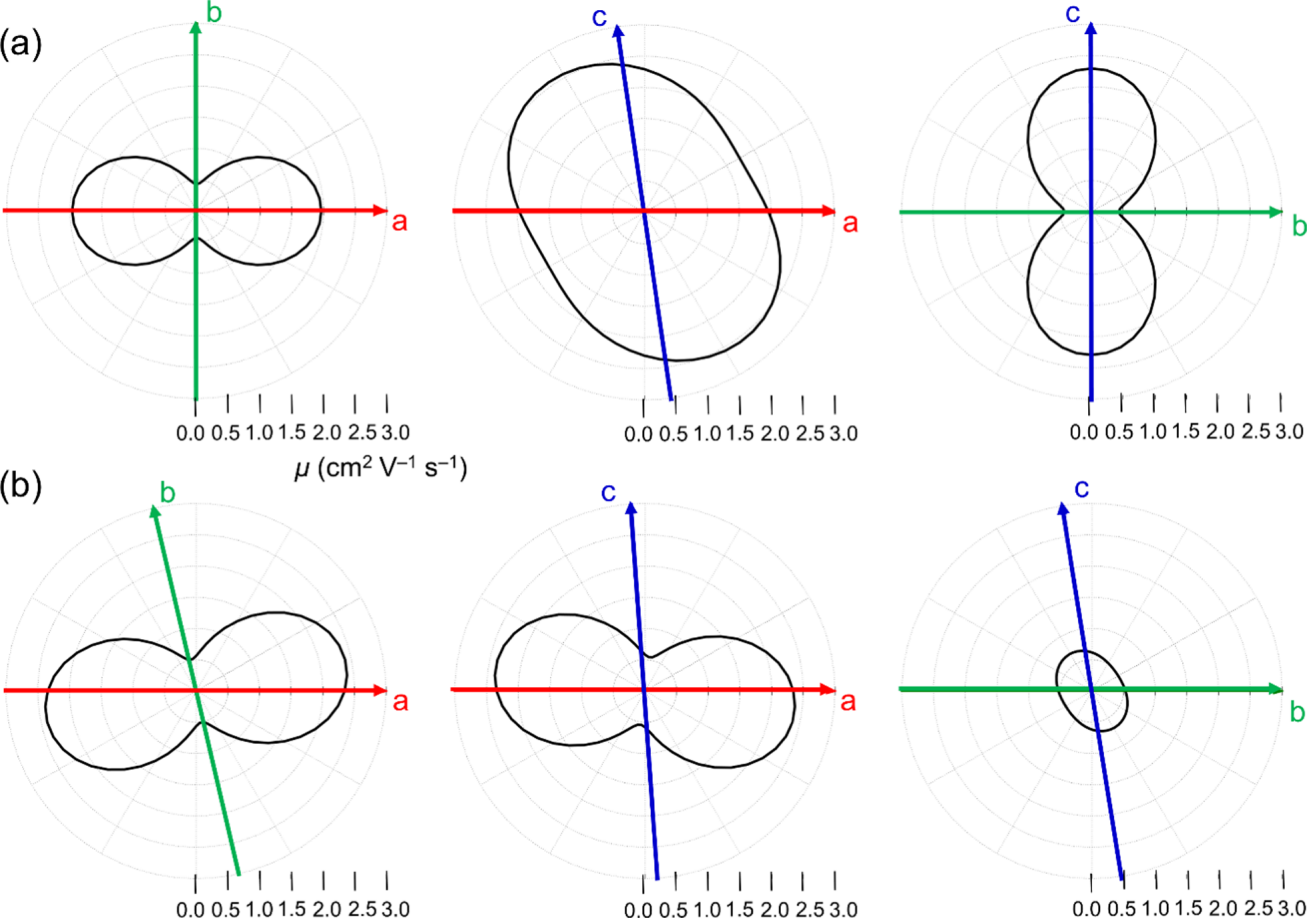
Hole mobilities (cm^2^ V^–1^ s^–1^) calculated for the **ADI** (a) and **ADAI** (b) crystal structures in the *ab* (left), *ac* (middle), and *bc* (right) crystallographic planes. The parameters used for the calculation were *F* = 1 × 10^5^ V cm^–1^ and *T* = 298 K.

Once all of the parameters involved in the MLJ rate expression are determined, the corresponding rates and, consequently, hole mobilities for the **ADI** and **ADAI** semiconductors can be estimated. To do so, a computational protocol based on analytic numerical methods was utilized (see the Supporting Information for further details).^72^Figure 5 shows the anisotropic hole mobilities calculated for the different crystallographic planes of the **ADI** and **ADAI** compounds. The predicted mobilities (∼0.5 to 2.5 cm^2^ V^–1^ s^–1^) are significantly overestimated with respect to the experimental measurements in line with other theoretical studies.^71^ Note that the calculations are performed using the crystal structures of **ADI** and **ADAI** with no defects and, thus, these values can be only used as an upper bound. In the thin films, the morphologies are expected to exhibit different crystalline domains and defects, which are not taken into account in the theoretical estimations. Nonetheless, the theoretical analysis offers insights into the intrinsic charge transport features of the **ADI** and **ADAI** crystals. Interestingly, both systems display significant mobilities in the three *ab*, *ac*, and *bc* crystallographic planes. Nevertheless, there are relevant differences: while **ADAI** exhibits an optimal charge transport direction along the *a* axis (π-stacked dimers), **ADI** displays a more isotropic transport in the *ab* plane in line with the presence of face-to-face and edge-to-face molecular pairs with significant *t*_*ij*_ values (Figure S4 and Table S1). Despite the difference in the charge transport anisotropy, both **ADI** and **ADAI** crystals have μ values in the same range for their optimal directions (∼2.5 cm^2^ V^–1^ s^–1^). The similarity in μ predicted for the **ADI** and **ADAI** crystals is seemingly in contrast to the experimental μ values estimated from the OFET devices, where **ADAI** showed higher mobilities than **ADI**. This experimental–theoretical discrepancy can be from morphological defects present in the thin films deposited for OFET fabrication. The hydrogen-bonded network present in **ADAI** produces more robust film morphologies (less disordered) and may explain the higher experimental mobilities observed for **ADAI** compared to **ADI**. This is further studied in the annealing experiments discussed below. Additionally, small relative differences in the orientation of neighboring molecules in the thin films compared to the single crystal, especially for π-stacked dimers in **ADAI**, can cause an enhancement in the charge transport owing to a more efficient orbital overlap (Figure S6).

### Material and Charge Transport Characterization upon Thermal Treatment

**Figure 6:**
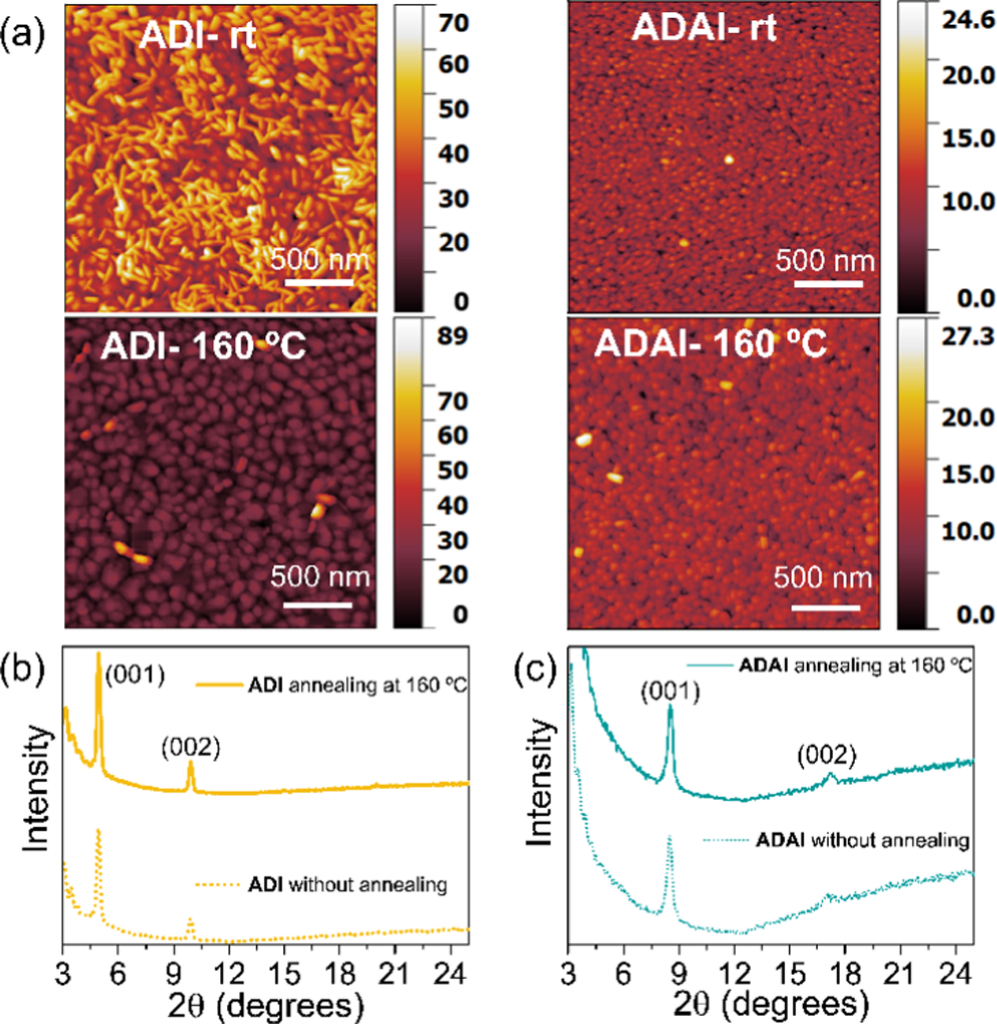
(a) Tapping mode AFM images. (b, c) XRD diffractograms before and after annealing.

Considering that the stability of the molecular materials is one of the critical issues to be solved in the area of organic electronics, we additionally studied the thermal robustness of the reported materials. The fabricated transistors were annealed at different temperatures following the evolution of their electrical performance and the thin-film morphology. Atomic force microscopy (AFM) images revealed that **ADAI** thin films showed a granular morphology (Figure 6). This type of microstructure has been previously correlated to good mobilities for similar molecules.^57^ This contrasts with the morphology observed for the material based on **ADI** that cannot form hydrogen bonds. Upon heating the substrate, while the **ADI** thin films experience significant changes in their microstructure, evolving to a granular morphology, the **ADAI** thin films remain virtually unchanged, with an increase in the grain size being displayed. This evidences the remarkable thermal robustness of the self-assembled material. Interestingly, X-ray diffraction measured on the annealed organic thin films confirmed that the diffractograms were essentially unchanged, although sharper peaks were detected (Figure 6b,c). Therefore, in spite of the morphological transformation observed for **ADI**, the crystal lattice was the same and it is likely that the crystalline domains grew larger. Although the sharpness of the diffraction peaks also increased in the case of **ADAI**, the difference is less pronounced.

**Figure 7:**
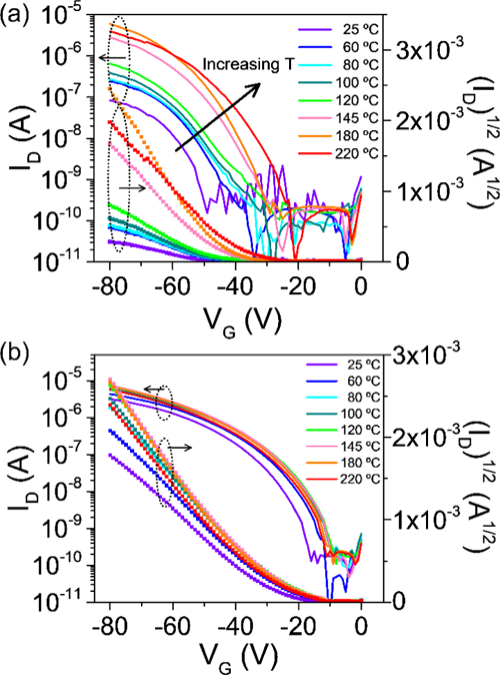
Transfer characteristics at different annealing temperatures for (a) **ADI** and (b) **ADAI**.

The characterization of the transistors during sequential annealing revealed noticeable changes in the **ADI** transfer characteristics, including a significant reduction of the switch-on voltage and an increase of mobility (Figure 7a). The thermal treatment also confirmed the robustness and high stability of **ADAI**, whose transfer characteristics displayed a very good reproducibility with a slight decrease in turn-on voltage and an increase in mobility (Figure 7b).

**Figure 8:**
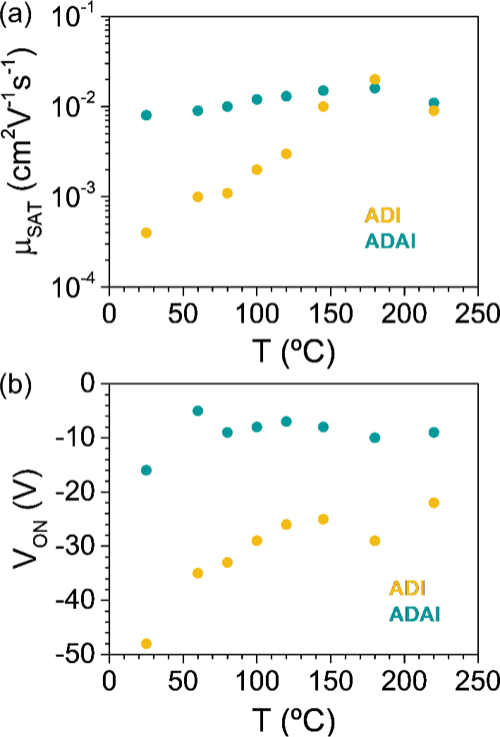
Evolution of (a) the saturated hole mobility (μ_SAT_) and (b) the switch-on voltage (*V*_ON_) with the annealing temperature.

Table S2 and Figure 8 summarize the OFET parameters for both materials measured at different annealing temperatures. The thermal lability of **ADI** results in improved mobility that reaches its best value after annealing at 180 °C, presumably due to the previously mentioned increase in the grain size and granular morphology. In contrast, the hydrogen-bonded structure confers stability to **ADAI**, which also improves its performance but with softer changes, keeping quite consistent higher mobilities throughout most of the annealing process. This would represent a practical advantage for a transistor fabrication process since no thermal treatment would be necessary for the processing of the self-assembled semiconductor. The similar mobilities observed for **ADI** and **ADAI** after annealing at more than 150 °C can result from the partial correction of the thin-film defects (especially in the case of the non-hydrogen-bonded **ADI**) and would be in agreement with the previously discussed similarities in the theoretical mobilities.

Finally, it is also interesting to analyze the evolution of the switch-on voltage upon annealing (Figure 8b). Several causes, mainly associated with processes occurring at the hybrid dielectric/organic semiconductor interface (SiO_2_-OTS/Semiconductor),^73^ are related to the shift of the switch-on voltage. The dipole orientation and/or dipolar disorder at the interface is correlated to the generation of charge traps. Moreover, charge trapping could also have its origin in the semiconductor itself, either at the interface or in the bulk, and is frequently associated with structural defects or disorder. In this regard, as corroborated by the present comparison, the hydrogen-bonded structure of **ADAI** can contribute to improving the nanostructural organization, attenuating the disorder in the semiconductor, and improving the transistor performance. Additionally, the semiconductor morphology also has a critical effect on the transistor parameters. For **ADI**, the thermal treatment causes an improvement in the transistor performance that is clearly associated with the morphological evolution toward a granular structure. This type of morphology is consistently observed for the self-assembled **ADAI** and results in a better device operation. It is worth noticing that the smaller grains of **ADAI** (average size 50 nm) enable a more compact packing between grains and better contact with the hybrid dielectric surface enhancing the transistor parameters, which is in line with other previously observed organic semiconductors.^74^ Conversely, the larger grains formed by **ADI** (average size 83 nm) and the space between grains seem to reduce the contact between the hybrid dielectric and the semiconductor, requiring a higher voltage to accumulate the charge carriers that lead to the transistor switch-on. Additionally, the lower thin-film roughness of **ADAI** (roughness_RMS_ at 160 °C: **ADAI** = 2.1 nm; **ADI** = 5.8 nm) is indicative of a less disordered material. In summary, these results reinforce the suitability of hydrogen bond-driven self-assembly for the development of novel organic semiconductors.

## Conclusions

We have synthesized two structurally related, pyrrole-based, fused heptacyclic molecules to investigate the effects that hydrogen bond-directed self-assembly has on the structural and electronic parameters governing charge transport. The use of 7-azaindole as a building block for π-conjugated systems has been proved to be a useful synthetic tool for the control of molecular organization through hydrogen bonding. While the non-self-assembled molecule shows a herringbone packing, the self-assembled material displays a slipped columnar packing stabilized by hydrogen bonds. Computational calculations have confirmed that this self-assembled columnar packing efficiently promotes charge transport with mobilities comparable to those for the common herringbone arrangement. Organic field-effect transistors fabricated with the hydrogen-bonded material exhibit better electrical performance in the form of lower switch-on voltages, higher charge carrier mobilities, and a weak dependence of the transfer characteristics on the annealing temperature. In contrast, OFETs made up with the analogous non-self-assembled material require significant thermal treatment to improve their performance. The thermal robustness of the hydrogen-bonded material is also supported by the characterization of the thin-film morphology that presents a granular structure and remains virtually unchanged upon increasing the temperature. In summary, we have demonstrated how molecular organization through hydrogen bonding is an effective strategy for the design of novel organic semiconductors with higher stability and robustness that can contribute to the progress of organic electronics.

## Supporting Information Available

ADI_structure (CIF)
^1^H NMR and ^13^C NMR spectra; TGA; theoretical results; and OFET parameters (PDF)

## Supplementary Material

am0c18928_si_001.cif
