# Supplementary material for: Improving the Robustness of Organic Semiconductors through Hydrogen Bonding
Source: ACS Appl Mater Interfaces. 2021 Feb 12;13(7):8620–30. doi: 10.1021/acsami.0c18928 (PMC8893359; doi:10.1021/acsami.0c18928)
Supplement: Supplementary file 2 — am0c18928_si_002.pdf [file am0c18928_si_002.pdf]

Supporting Information

Improving the Robustness of Organic Semiconductors through Hydrogen-Bonding

Paula Gómez,† Stamatis Georgakopoulos,† Miriam Más-Montoya,† Jesús Cerdá,‡ José Pérez,§ Enrique Ortí,‡ Juan Aragó,*,‡ and David Curiel*,†

† Multifunctional Molecular Materials Group, Department of Organic Chemistry, University of Murcia, Campus of Espinardo, 30100-Murcia, Spain.

‡ Institute of Molecular Science, University of Valencia, Catedrático José Beltrán 2, 46980 Paterna, Spain.

§ Department of Chemical Engineering and Environmental Chemistry, Regional Campus of International Excellence, Technical University of Cartagena, 30203-Cartagena, Spain.

Corresponding Authors:

* Dr. David Curiel. E-mail: davidcc@um.es

* Dr. Juan Aragó. E-mail: juan.arago@uv.es

NMR spectra S2

Thermal properties S4

Theory and computational results S4

OFETs parameters at different temperatures S9

NMR spectra

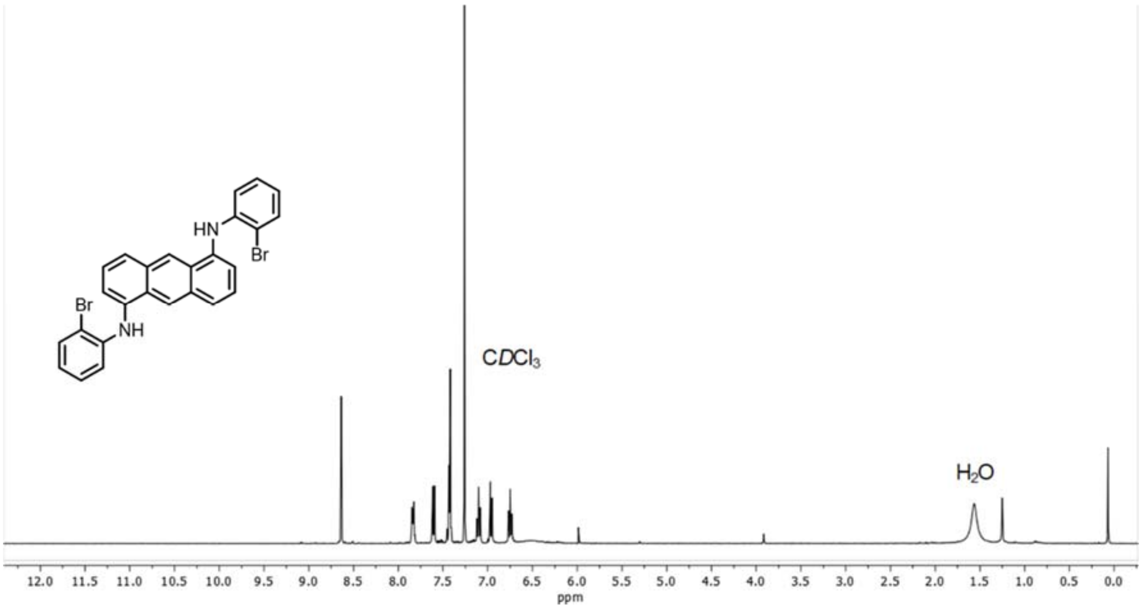

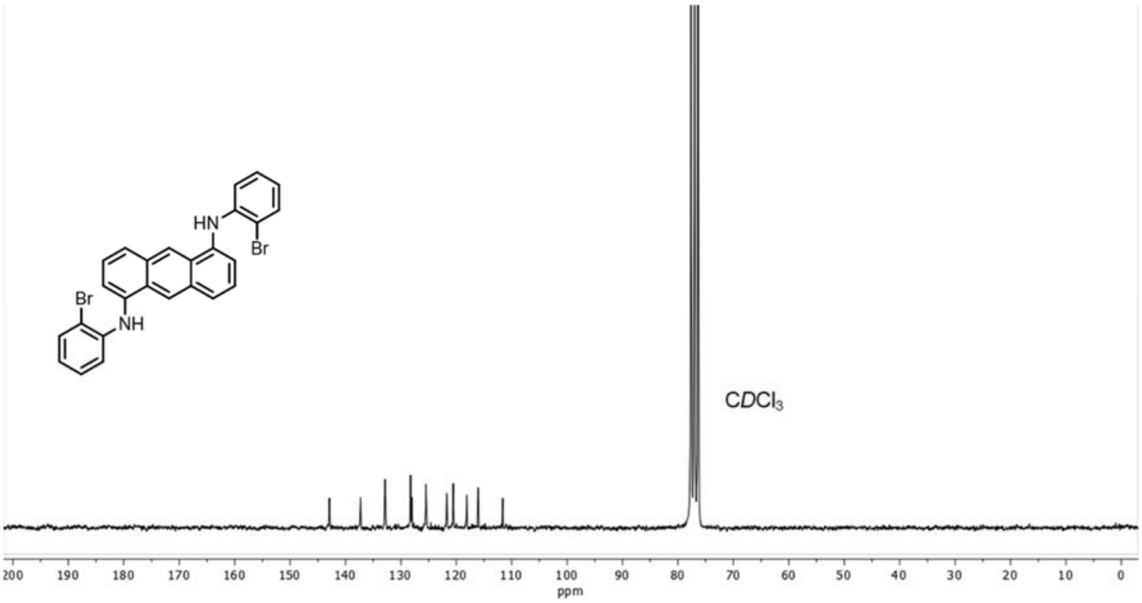

Figure S1. ^{1}H-NMR (400 MHz) and ^{13}C-NMR (50 MHz) spectra of compound **1** in $CDCl_3$.

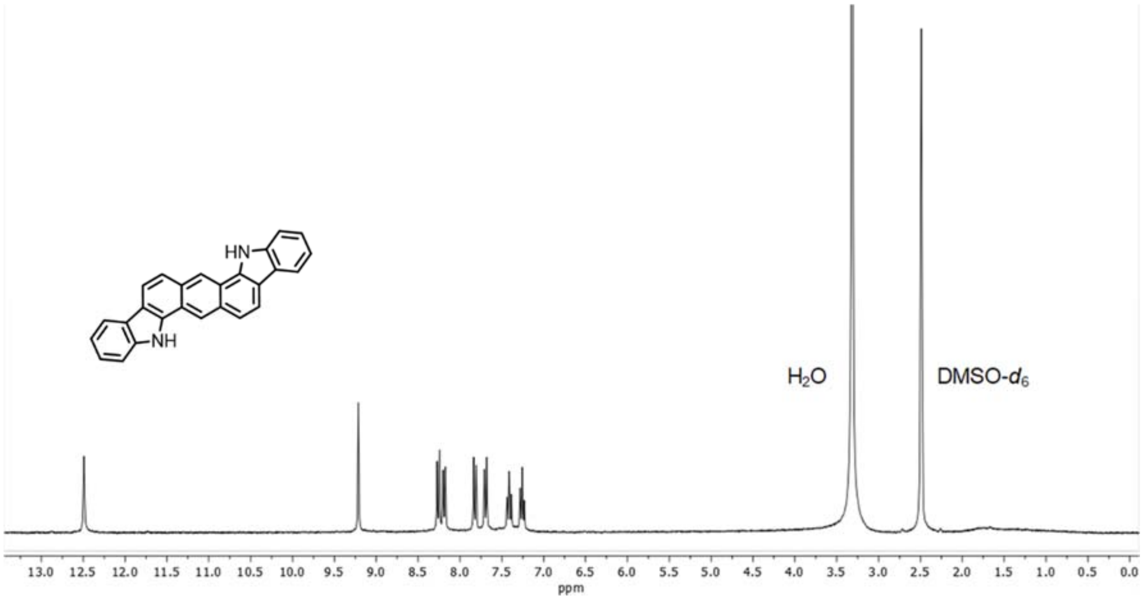

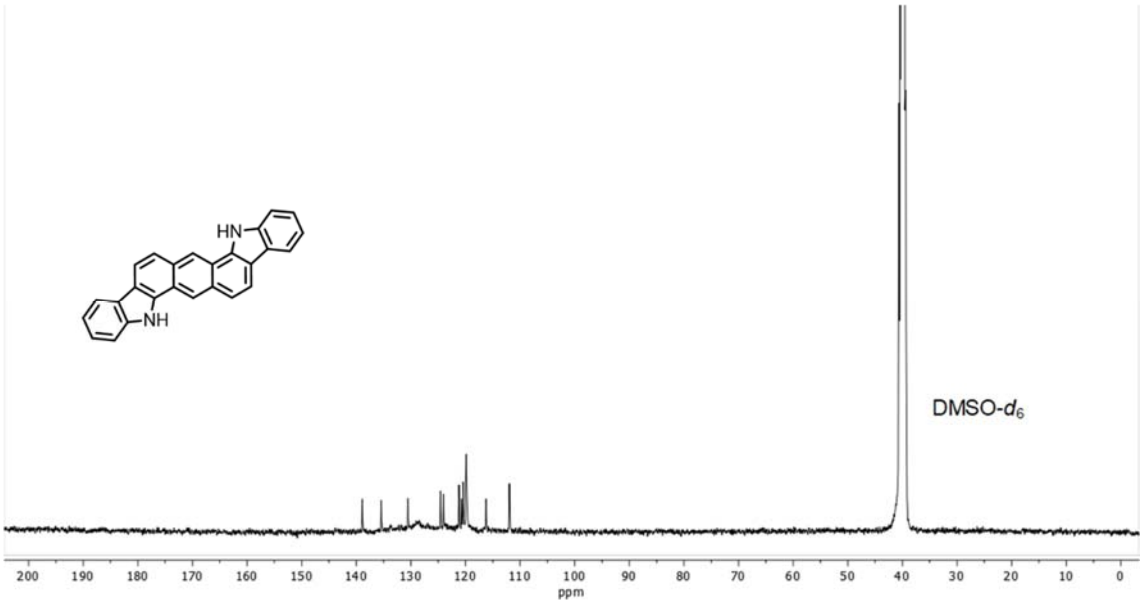

Figure S2. ^{1}H-NMR (300 MHz) and ^{13}C-NMR (100 MHz) spectra of **ADI** in DMSO-d_6.

Thermal properties

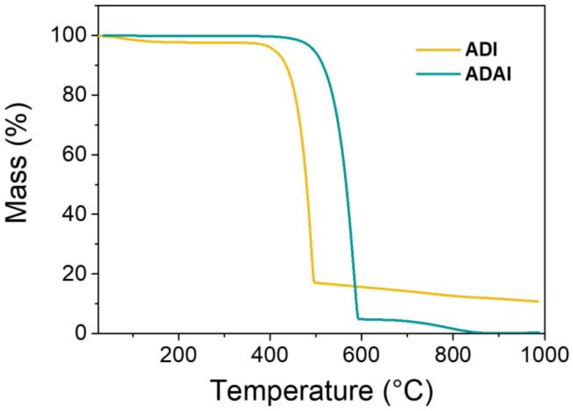

Figure S3. Thermogravimetric analysis.

Theory and computational results

Marcus–Levitch–Jortner (MLJ) Rate Constant and its Parameters**.** Within a hopping mechanism, a MLJ rate constant has been selected since the MLJ rate incorporates quantum tunneling effects through an effective vibrational normal mode coordinate. The non-adiabatic electron-transfer MLJ rate is expressed as follows:

$$k_{ij} = \frac{2\pi}{\hbar}\left\langle t_{ij}^2 \right\rangle \frac{1}{\sqrt{4\pi\lambda_{\text{ext}} k_B T}} \sum_n P_T(n) \sum_{n'} |FCI_{nn'}(S_{\text{eff}})|^2 \exp\left[-\frac{\left(\Delta E_{ij} + \lambda_{\text{ext}} + (n'-n)\hbar\omega_{\text{eff}}\right)^2}{4\lambda_{\text{ext}} k_B T}\right] \quad \text{(S1)}$$

where t_{ij} is the transfer integral between the hopping sites *i* and *j*. $\left\langle t_{ij}^2 \right\rangle$ incorporates the effect of the thermal fluctuation since $\left\langle t_{ij}^2 \right\rangle = \left\langle t_{ij} \right\rangle^2 + \sigma_{ij}^2$, where σ_{ij}^2 denotes the variance of t_{ij}. λ_{ext} is the external reorganization energy, k_B is the Boltzmann constant, *T* is the temperature (298 K) and $\hbar$ is the reduced Planck constant. ΔE_{ij} is the site energy difference, which in an ideal crystal structure is zero. Here, the energy difference ($\Delta E_{ij} = -q\vec{F}\vec{r}_{ij}$) is caused by an external electric field $\vec{F}$, where *q* is the charge (positive unit charge) and $\vec{r}_{ij}$ is the distance vector between sites *i* and *j*. $FCI_{nn'}(S_{eff})$ denotes the Franck−Condon integrals between the initial (*n*) and final (*n'*) vibrational levels between the initial *i* and final *j* molecular sites which have been calculated using an analytic expression under the harmonic approximation.[1] S_{eff} corresponds to the Huang–Rhys (HR) factor for an effective normal mode with frequency ω_{eff} . Finally, $P_T(n)$ represents the Boltzmann probability that a vibrational state *n* on an initial site *i* is occupied at a certain temperature.

The transfer integral t_{ij} (also known as electronic coupling) have been calculated according to the approach developed by Baumeier *et al.*,[2] in which the molecular orbitals of a dimer are projected on the basis of the molecular orbitals of the individual molecules.

To take into account the impact of the thermal motions on t_{ij}, molecular dynamics (MD) simulations (10 ps of equilibration and 100 ps of production) of cubic cells of 91 molecules for **ADI** and 125 molecules for **ADAI** were carried out at the GFN-FF level,[3] where the molecules in the edge of the crystal were kept frozen since periodic boundary conditions are not implemented in the xTB code. From the production simulation, 1000 snapshots with timesteps of 0.1 ps were extracted. For each snapshot, the transfer integrals t_{ij} for the most relevant dimers were calculated at the B3LYP/6-31G** level using a homemade program. Relevant dimers were considered to be those that displayed significant couplings (t_{ij} > 1 meV) at the experimental crystal structure (Table S1). Average transfer integral values ($\langle t_{ij} \rangle$) and their corresponding standard deviations ($\sigma_{t_{ij}}$) have been collected in Table S1.

The reorganization energy (λ) is a key parameter for the evaluation of electron transfer rates and is associated with the energy change owing to electronic redistribution and nuclear rearrangement in electron-transfer events.[4] Generally, λ is split into the internal and external reorganization components ($\lambda = \lambda_{\text{int}} + \lambda_{\text{ext}}$). The former is related to the energy cost due to the intramolecular nuclear relaxation of the molecular systems during the electron-transfer reaction whereas the latter comes from the environmental effects; *i.e.*, polarization and reorientation of neighboring molecules as a response of the charge (electron or hole) injection in a molecular system.

The internal reorganization energy λ_{int} has been estimated by using the four-point approach.[4] λ_{int} values of 159 and 183 meV have been calculated at the DFT level (B3LYP/6-31G**) for **ADI** and **ADAI**, respectively. λ_{int} has been also decomposed into contributions for each normal mode according to $\lambda_{\text{int}} = \sum_k \hbar\omega_k S_k$, where ω_k is the vibrational frequency of the normal mode k and S_k corresponds to the Huang−Rhys (HR) factor. The HR factors have been estimated according to Ref. 5. In the MLJ expression (Eq. S1), the rate constant is simplified because it makes use of only a single effective normal mode coordinate with frequency $\omega_{\text{eff}} = \sum_k \omega_k S_k / \sum_k S_k$ and an effective HR factor $S_{\text{eff}} = \lambda_{\text{int}} / \hbar\omega_{\text{eff}}$.[6] For **ADI** (**ADAI**), S_{eff} and ω_{eff} values of 1.235 (1.415) and 1036 cm^{-1} (1046 cm^{-1}) were obtained.

The external reorganization energy λ_{ext} for electron-transfer processes in molecular crystals is generally small[7] and is commonly neglected. Here, a common value of λ_{ext} = 50 meV has been set for both **ADI** and **ADAI** compounds in line with values reported for other similar systems.[8]

Master Equation and Mobilities. The charge transport can be described by solving a kinetic master equation. In the case of low charge-carrier densities, the master equation acquires the simple linear expression:

$$\frac{dp_i}{dt} = \sum_j \left(k_{ij} p_j - k_{ji} p_i \right), \tag{S2}$$

where p_i corresponds to the probability that the site i is occupied by a charge (hole/electron) carrier. When the dynamic balance is reached (steady state), the occupation probabilities for the sites do not change anymore ($dp_i/dt = 0$). Eq. S2 can be rewritten in a matrix form as

$$\vec{0} = \vec{p}\ \mathbf{N}, \tag{S3}$$

where $\vec{p}$ contains the unknown p_i and **N** is a negative semidefinite sparse matrix that collects all electron-transfer rates k_{ij}. **N** is approximated by a finite matrix by applying cyclic boundary conditions. In this work, a crystal super-cell model (4 × 4 × 4) of grid points corresponding to the molecular centroids of the **ADI** (128 sites) and a 5 × 5 × 5 super-cell (125 sites) for **ADAI** molecules were used to solve Eq. S3. Once all the p_i probabilities are obtained and the normalization condition ($\sum_i p_i = 1$) has taken into account, the hole mobility in the external electric field direction is calculated according to:

$$\mu = \frac{1}{F} \sum_{ij} p_i k_{ij} \vec{r}_{ij} \frac{\vec{F}}{F} \tag{S4}$$

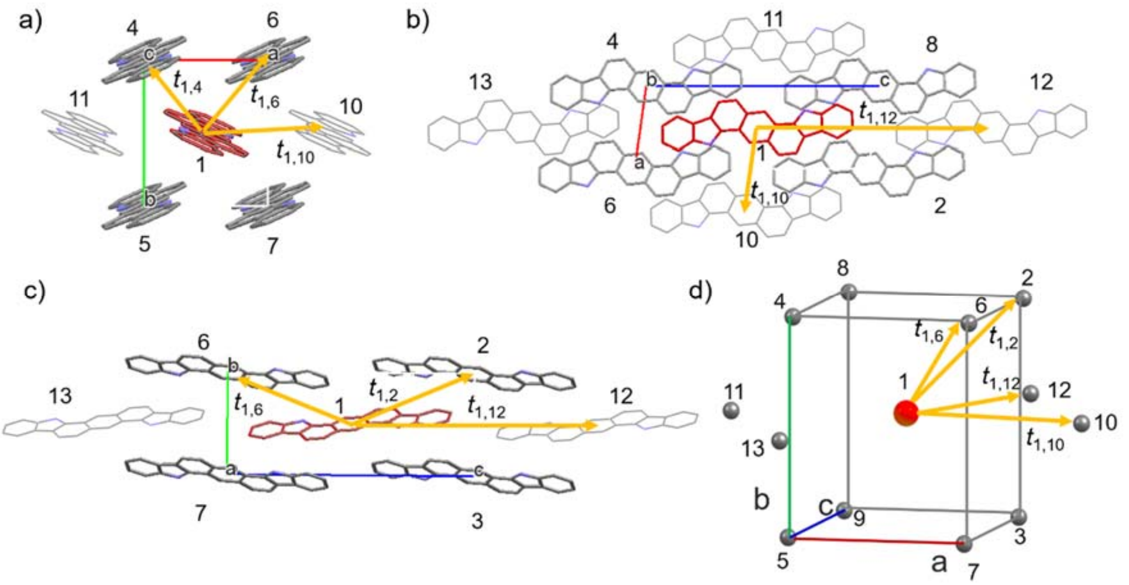

Figure S4. Crystal structure views of the *ab* (a), *ac* (b), and *bc* (c) crystallographic planes of **ADI**. d) Grid representation of the crystal unit cell of **ADI**, where all different and non-negligible t_{ij} values starting from the central molecule are shown (see Table S1). H atoms have been omitted for simplicity.

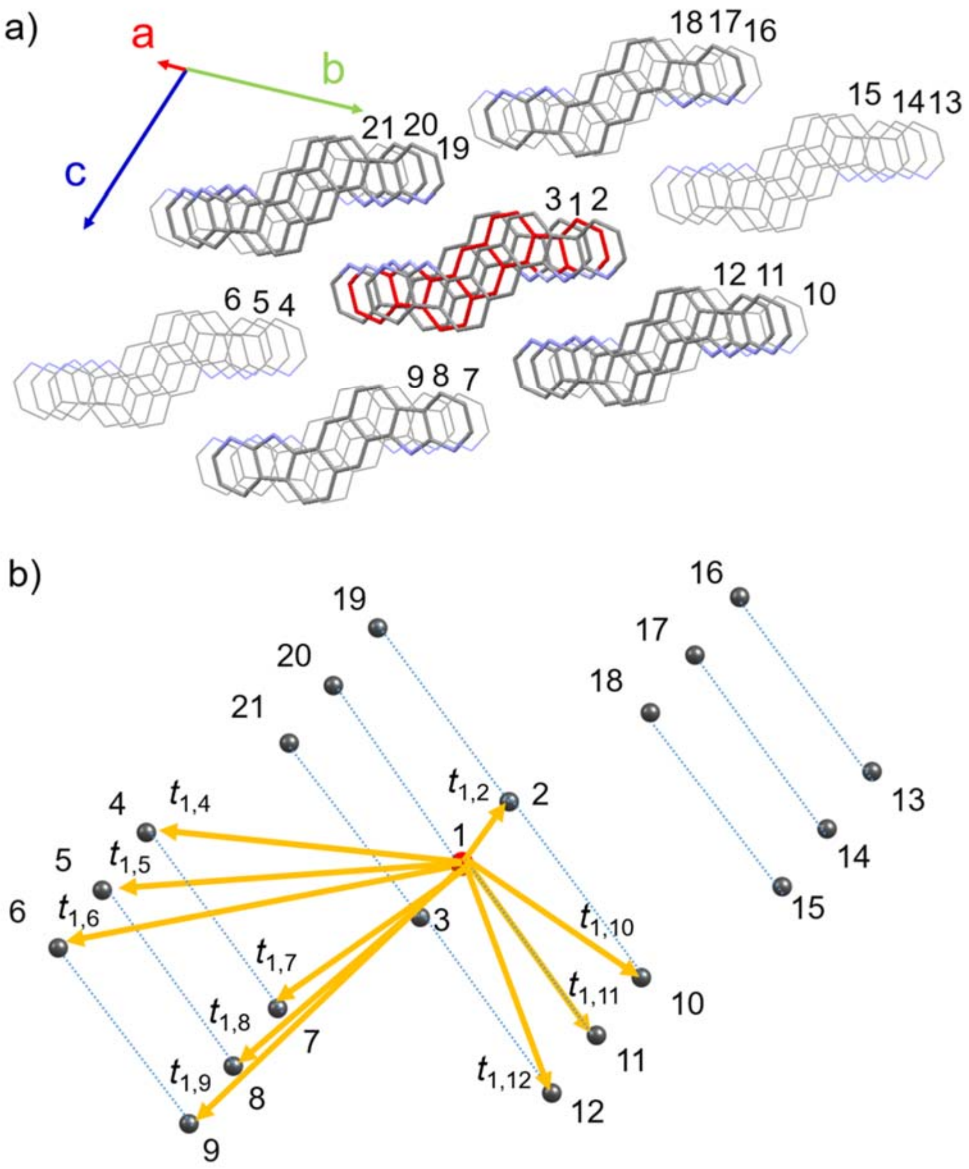

Figure S5. a) Crystal structure of **ADAI**. b) Grid representation of the crystal structure shown in (a) where all different and non-negligible t_{ij} values involving the central molecule are shown (see Table S1). H atoms have been omitted for simplicity. Dotted blue lines indicates the molecules that form H-bonds.

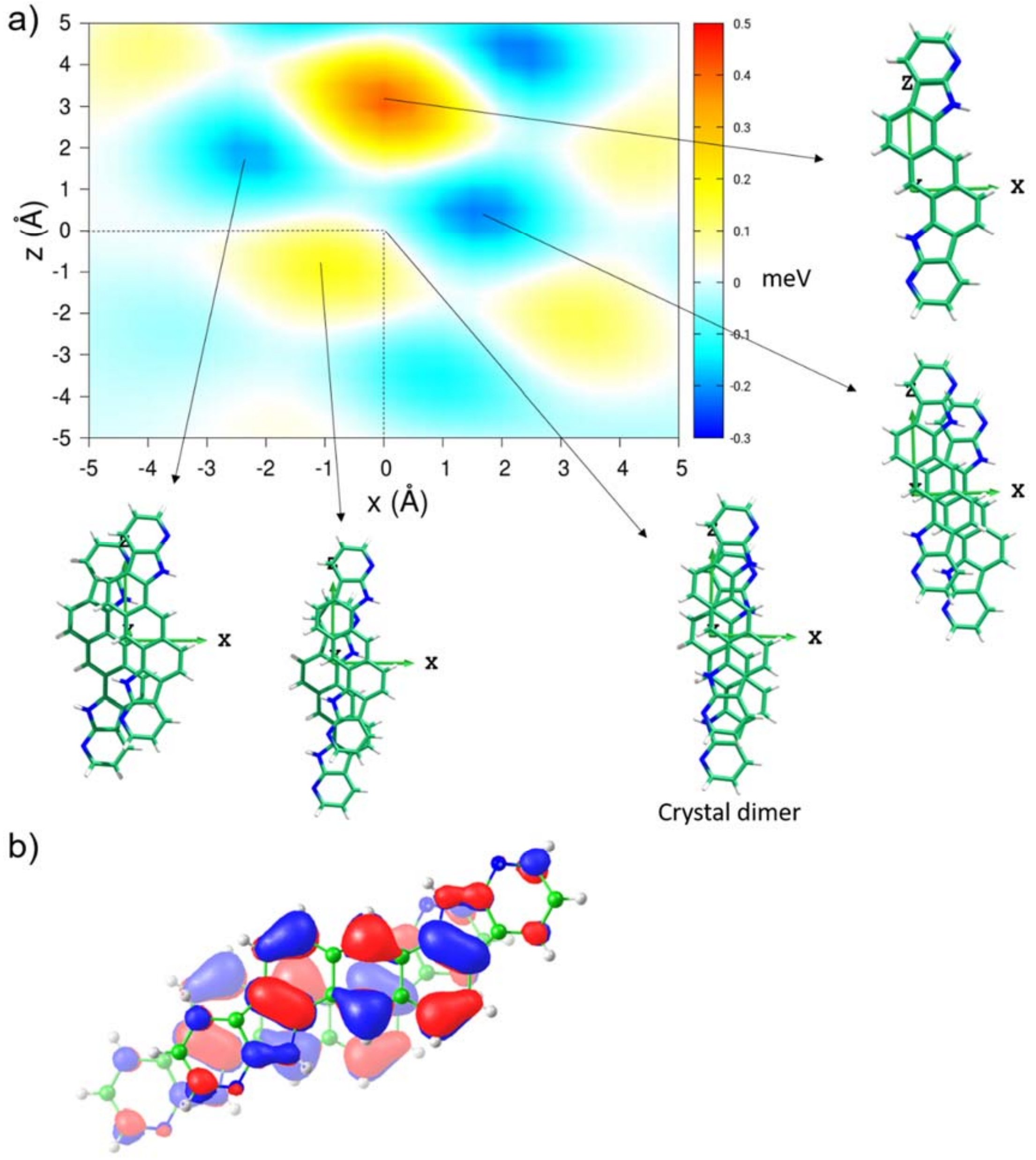

Figure S6. a) 2D map of t_{ij} (in meV) for a face-to-face π-stacked dimer of **ADAI** calculated by moving one of the molecules along the *x* and *z* directions starting from the crystal structure (*x* = 0 Å and *z* = 0 Å). b) Topologies of the HOMOs for a face-to-face π-stacked dimer of **ADAI** at the crystal structure (top and bottom molecules are represented with bright and faded colors, respectively).

Table S1. Average transfer integrals ($\langle t_{ij} \rangle$) and standard deviations ($\sigma_{t_{ij}}$) in meV calculated for the different molecular pairs of **ADI** and **ADAI** crystals from MD simulations (300K) (Figure S4 and S5). t_{ij} corresponds to transfer integrals computed at the experimental crystal structure. Transfer integrals were computed at the B3LYP/6-31G** level. The t_{ij} values for the face-to-face π-stacked dimers in both **ADI** and **ADAI** are highlighted in blue.

**ADI**				**ADAI**			
Different t_{ij}	t_{ij}	$\langle t_{ij} \rangle$	$\sigma_{t_{ij}}$	Different t_{ij}	t_{ij}	$\langle t_{ij} \rangle$	$\sigma_{t_{ij}}$
$t_{1,2} = t_{1,3} = t_{1,4} = t_{1,5}$	14.4	–6.8	16.9	$t_{1,2} = t_{1,3}$	11.1	0.0	57.8
$t_{1,6} = t_{1,7} = t_{1,8} = t_{1,9}$	6.7	–8.1	10.6	$t_{1,4} = t_{1,15}$	0.7	-	-
$t_{1,10} = t_{1,11}$	40.0	37.7	14.5	$t_{1,5} = t_{1,14}$	0.4	-	-
$t_{1,12} = t_{1,13}$	0.3	-	-	$t_{1,6} = t_{1,16}$	0.0	-	-
				$t_{1,7} = t_{1,18}$	0.2	-	-
				$t_{1,8} = t_{1,17}$	12.1	–12.3	6.5
				$t_{1,9} = t_{1,16}$	0.4	-	-
				$t_{1,10} = t_{1,21}$	0.2	-	-
				$t_{1,11} = t_{1,20}$	8.4	3.0	2.6
				$t_{1,12} = t_{1,19}$	16.7	–13.3	10.0

OFETs parameters at different temperatures

Table S2. OFETs parameters.

	ADI			ADAI		
T (°C)	μ [cm² V⁻¹ s⁻¹]	V_{on} [V]	I_{on}/I_{off}	μ [cm² V⁻¹ s⁻¹]	V_{on} [V]	I_{on}/I_{off}
25	$4.0\times10^{-4} \pm 1\times10^{-4}$	−48 ± 1	5×10^{2}	$7.9\times10^{-3} \pm 4\times10^{-4}$	−16 ± 3	2×10^{4}
60	$1.0\times10^{-3} \pm 2\times10^{-4}$	−35 ± 4	3×10^{4}	$9.0\times10^{-3} \pm 3\times10^{-4}$	−5 ± 1	2×10^{6}
80	$1.1\times10^{-3} \pm 1\times10^{-4}$	−33 ± 4	4×10^{4}	$1.0\times10^{-2} \pm 2\times10^{-3}$	−9 ± 3	6×10^{4}
100	$1.9\times10^{-3} \pm 5\times10^{-4}$	−29 ± 3	3×10^{5}	$1.2\times10^{-2} \pm 2\times10^{-3}$	−8 ± 2	4×10^{4}
120	$3.1\times10^{-3} \pm 3\times10^{-4}$	−26 ± 3	6×10^{3}	$1.3\times10^{-2} \pm 2\times10^{-3}$	−7 ± 2	6×10^{4}
145	$1.0\times10^{-2} \pm 2\times10^{-3}$	−25 ± 3	2×10^{5}	$1.5\times10^{-2} \pm 1\times10^{-3}$	−8 ± 2	5×10^{4}
180	$2.0\times10^{-2} \pm 3\times10^{-3}$	−29 ± 3	5×10^{4}	$1.6\times10^{-2} \pm 1\times10^{-3}$	−10 ± 2	7×10^{4}
220	$9.0\times10^{-3} \pm 1\times10^{-3}$	−22 ± 2	4×10^{5}	$1.1\times10^{-2} \pm 2\times10^{-3}$	−9 ± 2	3×10^{4}

Average values and standard deviations obtained from six devices.

References

[1] May, V.; Khün, O. *Charge and Energy Transfer Dynamics in Molecular Systems*, third edit.; WILEY-VCH Verlag: Weinheim, Germany, 2011.

[2] Baumeier, B.; Kirkpatrick, J.; Andrienko, D. Density-Functional Based Determination of Intermolecular Charge Transfer Properties for Large-Scale Morphologies. *Phys. Chem. Chem. Phys.* **2010**, *12*, 11103.

[3] Spicher, S.; Grimme, S. Robust Atomistic Modeling of Materials, Organometallic, and Biochemical Systems. *Angew. Chem. Int. Ed.* **2020**, *59*, 15665–15673.

[4] Coropceanu, V.; Cornil, J.; da Silva Filho, D. A.; Olivier, Y.; Silbey, R.; Brédas, J.-L. Charge Transport in Organic Semiconductors. *Chem. Rev.* **2007**, *107*, 926–952.

[5] Malagoli, M.; Coropceanu, V.; da Silva Filho, D. A.; Brédas, J. L. A Multimode Analysis of the Gas-Phase Photoelectron Spectra in Oligoacenes. **2004**, *120*, 7490–7496

[6] Troisi, A., Charge transport in high mobility molecular semiconductors: classical models and new theories. *Chem. Soc. Rev. 2***011,** 4*0,* 2347-2358.

[7] (a) Norton, J. E.; Brédas, J.-L. Polarization Energies in Oligoacene Semiconductor Crystals. *J. Am. Chem. Soc.* **2008**, *130*, 12377–12384. (b) McMahon, D. P.; Troisi, A. Evaluation of the External Reorganization Energy of Polyacenes. *J. Phys. Chem. Lett.* **2010**, *1*, 941–946.

[8] Stehr, V.; Fink, R. F.; Tafipolski, M.; Deibel, C.; Engels, B. Comparison of Different Rate Constant Expressions for the Prediction of Charge and Energy Transport in Oligoacenes. *Wiley Interdiscip. Rev. Comput. Mol. Sci.* **2016**, *6*, 694–720.
